# Differential Expression of Prostaglandin I2 Synthase Associated with Arachidonic Acid Pathway in the Oral Squamous Cell Carcinoma

**DOI:** 10.1155/2018/6301980

**Published:** 2018-11-08

**Authors:** Anelise Russo, Patrícia M. Biselli-Chicote, Rosa S. Kawasaki-Oyama, Márcia M. U. Castanhole-Nunes, José V. Maniglia, Dalísio de Santi Neto, Érika C. Pavarino, Eny M. Goloni-Bertollo

**Affiliations:** ^1^Department of Molecular Biology: Biological and Genetics and Molecular Biology Research Unit – UPGEM, São José do Rio Preto Medical School – FAMERP, São José do Rio Preto, SP 15090-000, Brazil; ^2^Department of Otorhinolaryngology and Head and Neck Surgery, FAMERP, São José do Rio Preto, SP 15090-000, Brazil; ^3^Department of Pathology, FAMERP, São José do Rio Preto, SP 15090-000, Brazil

## Abstract

**Introduction:**

Differential expression of genes encoding cytochrome P450 (CYP) and other oxygenases enzymes involved in biotransformation mechanisms of endogenous and exogenous compounds can lead to oral tumor development.

**Objective:**

We aimed to identify the expression profile of these genes, searching for susceptibility biomarkers in oral squamous cell carcinoma.

**Patients and Methods:**

Sixteen oral squamous cell carcinoma samples were included in this study (eight tumor and eight adjacent non-tumor tissues). Gene expression quantification was performed using TaqMan Array Human CYP450 and other Oxygenases 96-well plate (Applied Biosystems) by real time qPCR. Protein quantification was performed by ELISA and IHC methods. Bioinformatics tools were used to find metabolic pathways related to the enzymes encoded by differentially expressed genes.* Results. CYP27B1, CYP27A1, CYP2E1, CYP2R1, CYP2J2, CYP2U1, CYP4F12, CYP4X1, CYP4B1, PTGIS, ALOX12,* and* MAOB* genes presented differential expression in the oral tumors. After correction by multiple tests, only the* PTGIS (Prostaglandin I2 Synthase)* gene presented significant differential expression (P < 0.05). The PTGIS gene and protein were reduced in oral tumors.

**Conclusion:**

PTGIS presents downexpression in oral tumors. PTGIS play an important role in the arachidonic acid metabolism. Arachidonic acid and/or metabolites are derived from this pathway, which can influence the regulation of important physiological mechanisms in tumorigenesis process.

## 1. Introduction

Worldwide, approximately 600,000 new cases of head and neck cancer HNC are diagnosed each year, and oral cancer comprises about half of these cases and is considered the most representative site of this tumor type [1]. In Brazil, the National Cancer Institute (INCA) estimates for each year of the biennium 2018-2019 that there are, 14,700 new cases of oral cavity cancer, representing the fifth-most-common cancer in men with 11,200 new cases. Regarding females, it was estimated that there are 3,500 new cases [2].

Literature data also show a high prevalence of oral cancer, which has been attributed to oral sex leading to increased infection with human papilloma virus (HPV) [3, 4]. This virus can be related to oxidative stress changes in saliva [5] as a reduction of antioxidant mechanism, increased reactive oxygen species (ROS) [6], and reactive nitrogen species (RNS), which leads to damage to DNA [7–9]. These changes prevent the antioxidative system of saliva from exercising its protective function, allowing carcinogenic compounds to act in the oral cavity [5]. Xenobiotic compounds such as N-nitrosamines, polycyclic aromatic hydrocarbons (HPAs) from tobacco [10, 11], and acetaldehyde, the primary metabolite of alcohol [12, 13], bind to DNA to form stable adducts [9].

Genes encoding enzymes involved in the activation mechanism and subsequent detoxification of carcinogenic compounds can present genetic polymorphisms [8, 14–20] that can modulate the gene expression, [21] leading to cancer development [21, 22].

CYP450 enzymes are monooxygenases functionally related to other oxygenases, such as monoamine oxygenase and the lipoxygenase family [23, 24]. In addition to participating in the pathway of xenobiotics, monooxygenases catalyze reactions in which a hydroxyl group is added to the protein, lipid, or other ligand. Members of this family participate in both biosynthesis and degradation of steroids, vitamins, fatty acids, arachidonic acid, prostaglandins, amines, pheromones, and plant metabolites [21, 24–26]. They also metabolize several drugs and chemical carcinogens/mutagens, among other environmental pollutants denominated as xenobiotics [27–29].

Thus, enzymes involved in xenobiotic biotransformation pathways can participate indirectly in the carcinogenesis mechanism due to their major role in individual susceptibility to disease, as they are responsible for the activation and detoxification of these compounds [21, 22]. For this reason, studies have been conducted to verify the association between the expression of genes encoding those enzymes and oral squamous cell carcinoma (OSCC) [30, 31], which accounts for 90% of malignant oral cancers [32]. However, there are very few studies on genome-wide profiling of OSCC tumors.

Considering the evidence presented, this study was designed to investigate the expression pattern of genes and proteins of the CYP450 family and other oxygenases involved in the biotransformation mechanism of endogenous compounds and xenobiotics in OSCC and compare them to adjacent non-tumor tissues. Moreover, the study was designed to identify the metabolic pathways related to genes differentially expressed in OSCC, enabling establishment of the importance of these genes in the carcinogenesis of OSCC.

## 2. Patients and Methods

This study is in accordance with the regulations of Resolution 466/12 of the National Health Council and was approved by the Committee of Ethics in Research–São José do Rio Preto Medical School (CEP-FAMERP), No. 216,758.

### 2.1. Samples Characterization

After informed consent, eight samples of tumor tissue and the adjacent non-tumor tissues of patients with OSCC coming from Otorhinolaryngology and Head and Neck Surgery of Hospital Base, Complex FAMERP/FUNFARME, were included in the study.

Inclusion criteria of samples in the study were pathological confirmation of primary tumor tissue of OSCC and adjacent non-tumor tissue, negative diagnosis for HPV 16 and 18 types, and sufficient concentration for quantification of gene expression. The exclusion criteria were tumor from relapse or patients treated with radiotherapy and/or chemotherapy before surgery.

Tumors were classified by the medical team participant of this study according to the parameters of Oncology Manual, Union for International Control Cancer (UICC), and the American Joint Committee for Cancer (AJCC) [33–35] on three criteria: tumor extension (T), nodal metastasis (N), and distant metastasis (M). T classification was divided into smaller (T1, T2) and larger (T3, T4) tumors. The N classification was defined as absence (N0) and presence (N1, N2, N3) of nodal metastasis. M classification was dichotomized in absence (M0) or presence (M1) of distant metastasis. The stages were divided into early (stages I and II) and advanced disease (III and IV) [33–35]. The diagnosis, primary tumor site, TNM classification, and some clinical information were obtained from medical records of the patients included in the study.

The study consisted of male patients with mean age of 68.25 years (± 10.30). Regarding the TNM, 50% of the tumors presented larger extension, 62.5% had nodal metastasis, and 62.5% had an absence of distant metastasis. In relation to tumor progression, 37.5% of cases were classified as non-advanced tumors (stages I and II) and 62.5% of cases as advanced tumors (stages III and IV).

### 2.2. Molecular Analysis

OSCC and adjacent non-tumor tissues were immediately stored in liquid nitrogen for transport and subsequently stored in a freezer at -80°C until processing. Genomic DNA and total RNA were extracted by sequential extraction methodology with TRIzol® Reagent (Ambion, Austin, TX), and the extracted total RNA was purified using the mirVana™ PARIS™ Kit (Applied Biosystems, Foster City, CA), according to manufacturer instructions.

According to literature data, the HPV16 and HPV18 types, which present E6 and E7 oncogenes, can participate in oral cavity carcinogenesis [36–40]. The detection of HPV16 and HPV18 by real-time Polymerase Chain Reaction (PCR) was performed from genomic DNA in collaboration with the “Instituto de Medicina Tropical de São Paulo–Universidade de São Paulo (USP)” [41, 42].

The purified total RNA samples were submitted to Reverse Transcription-Polymerase Chain Reaction (RT-PCR) for complementary DNA (cDNA) synthesis using the High-Capacity cDNA Reverse Transcription® (Applied Biosystems, Foster City, CA) according to manufacturer instructions.

In the present study, the standard curve was performed with GAPDH and HPRT1 reference genes to analysis of efficiency and determine the amount of sample to be used in the reactions.

**Figure 1:**
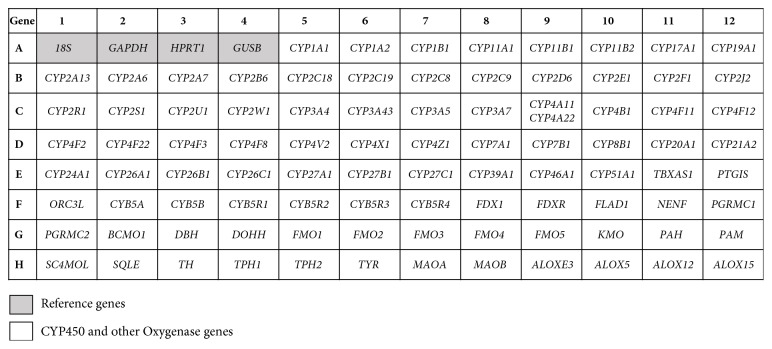
Graphical representation of the TaqMan® Array Human CYP450 and other Oxygenases 96-well Plate.

Gene expression quantification of genes involved in biotransformation mechanism of endogenous and exogenous compounds was performed using the TaqMan® Array Human CYP450 and other Oxygenases, Fast 96-well Plate (Applied Biosystems, Foster City, CA–catalogue number 4418730) by quantitative real-time PCR (qPCR). The panel of assays designed by the manufacturer allowed the evaluation of four reference genes (RNA, 18S ribosomal (18S), glyceraldehyde-3-phosphate dehydrogenase (GAPDH), hypoxanthine phosphoribosyltransferase 1 (HPRT1), and glucuronidase, beta (GUSB)) as endogenous controls, 92 genes of the CYP450 family, and other oxygenases such as monoamine oxygenases and lipooxygenases (Figure 1). The reactions were performed in duplicate with 25ng of cDNA on StepOnePlusTM Real-Time PCR System (Applied Biosystems, Foster City, CA) and cycled at 50°C for 2 minutes, 95°C for 10 minutes followed by 40 cycles at 95°C for 15 seconds and 60°C for 1 minute.

Raw qPCR data (quantification cycle values) were calculated by ExpressionSuite software version 1.0.3 (Applied Biosystems, Foster City, CA) after manual adjustment of the basal fluorescence signal (baseline) and the threshold for each gene analyzed.

For normalization of gene quantification data, the geometric mean of multiple reference genes was used [43]. The calculation of relative quantification (RQ) was performed, using samples of non-tumor tissue as calibrators, by the 2^−ΔΔCq^ method [44–46].

### 2.3. Bioinformatic Analysis of Differentially Expressed Genes

The bioinformatics tools the Kyoto Encyclopedia of Genes and Genomes (KEGG) [47], the Search Tool for the Retrieval of Interacting Genes/Proteins Information (STRING) [48], the National Center for Biotechnology (NCBI) [49], GeneCards®: The Human Gene Database [50], “Descritores em Ciências da Saúde” (DeCS) [51], Protein knowledgebase (UniProtKB) [52], and European Molecular Biology Laboratory–The European Bioinformatic Institute (EMBL–EBI) [53] were used to investigate the biological functions and metabolisms in which the enzymes encoded by differentially expressed genes are involved.

### 2.4. Protein Quantification

Among the differentially expressed genes, five were selected (CYP2E1, CYP2J2, CYP2U1, ALOX12B, and PTGIS) for protein quantification. For protein extraction, OSCC and adjacent non-tumor fragment tissues were washed with Phosphate Buffered Saline (PBS) 1X at 7.0–7.2 pH. Subsequently, the fragment was shredded into small pieces in a PBS1X buffer and taken to the sonicator with pulse 3 for 30 seconds in 3 cycles, with an interval of 1 minute between cycles, followed by centrifugation of 5,000 rpm for 15 minutes at 4°C. The proteins were quantified by BCA kit (Thermo Fisher) in a microplate reader at 562nm filter.

### 2.5. Enzyme-Linked Immunosorbent Assay (ELISA)

Comparison of protein concentration between tumors and non-tumor tissues was performed by Enzyme-Linked Immunosorbent Assay (ELISA) using CYP2E1, CYP2J2, CYP2U1, ALOX12B, and PTGIS BioAssay™ ELISA kit (United States Biological, Life Science Massachusetts, USA) according to the manufacturer instructions. Reactions were performed using 10 ug of total protein and quantified in a microplate reader at 450nm filter.

### 2.6. Immunohistochemistry (IHC)

Immunohistochemistry was carried out on three-micrometer thick sections of the OSCC tumors paraffin blocks. Non-tumor tissue margins were used as reference expressions.

Immunohistochemistry analysis was possible for ALOX12B and PTGIS proteins using the REVEAL Biotin-Free Detection System (Spring Bioscience, Pleasanton, CA) with primary antibodies: Anti-PTGIS antibody ab23668 (dilution 1:500; Abcam, Cambridge, UK) and ALOX12B antibody NBP1-89409 (dilution 1:500; Novus biologicals, Colorado, USA) incubated overnight at 4°C. For ALOX12B, after the endogenous peroxidase blocking step, the tissue permeabilization was carried out, in which washes were performed in 1X PBS with 0.025% of Triton X-100 for 10 minutes at room temperature. At the end, the slides were mounted in Entellan (Merck, Darmstadt, Germany).

Slides with OSCC and non-tumor tissues were photographed, and protein expression was evaluated by the average of score values from 0 to 4 (score 0: 0–5%, score 1: 5–25%, score 2: 25–50%, score 3: 50–75% and score 4: 75–100%) according to staining.

### 2.7. Statistical Analysis

The D'Agostino & Pearson omnibus normality test was performed to evaluate normal distribution of the data. Relative gene quantification was performed by One-sample T test or Wilcoxon signed rank test. Correction for multiple tests described by Benjamini and Hochberg (1995) (Benjamini-Hochberg False Discovery Rate) was applied to correct the occurrence of false positives [54]. ELISA and Immunohistochemistry data were evaluated by T test, Mann Whitney test, or Wilcoxon matched-paired test. The correlation between gene expression and protein expression values was conducted by Pearson or Spearman correlation test. p values < 0.05 were considered significant, and statistical analyses were performed using GraphPad Prism v.5 and StatsDirect v.2.7.2 programs.

## 3. Results

**Figure 2:**
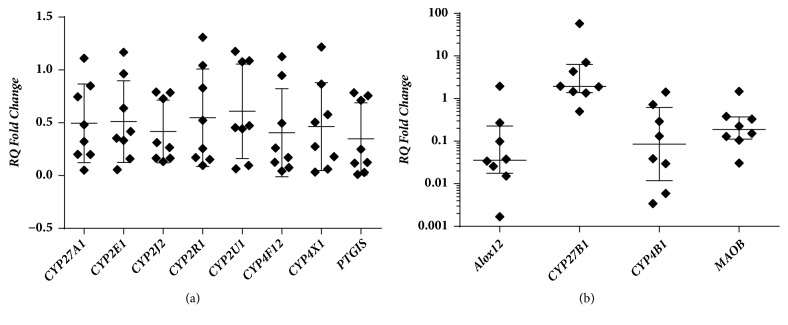
Gene expression in oral tumors in relation to non-tumor tissues. (a) Genes analyzed by One Sample T Test (mean with standard deviation). The RQ values are presented in linear scale. (b) Genes analyzed by Wilcoxon signed rank test (median with interquartile variation). The RQ values are shown in base 10 logarithmic scale.

Our results showed that 12 genes showed differential expression in OSCC tissues compared to adjacent non-tumor tissues (*P *< 0.05). The* CYP27B1* gene showed overexpression in OSCC, while* CYP27A1*,* CYP2E1, CYP2R1, CYP2J2, CYP2U1, CYP4F12, CYP4X1, CYP4B1, PTGIS, ALOX12B, *and* MAOB* genes showed reduced expression (Table 1 and Figure 2). After correction for multiple tests of the Benjamini-Hochberg False Discovery Rate, the* PTGIS *gene showed significantly different expression levels.

The 12 differentially expressed genes were related to 24 metabolic pathways of the Human species (Table 2). Among them, 12 pathways were associated with the carcinogenesis process, highlighting the arachidonic acid metabolism, in which five of the differentially expressed genes in OSCC (*ALOX12*,* CYP2E1*,* CYP2J2*,* CYP2U1,* and* PTGIS*) act. Because the role of arachidonic acid metabolism was representative in this study, the proteins encoded by these genes were selected for protein analysis by ELISA and IHC [47].

**Figure 3:**
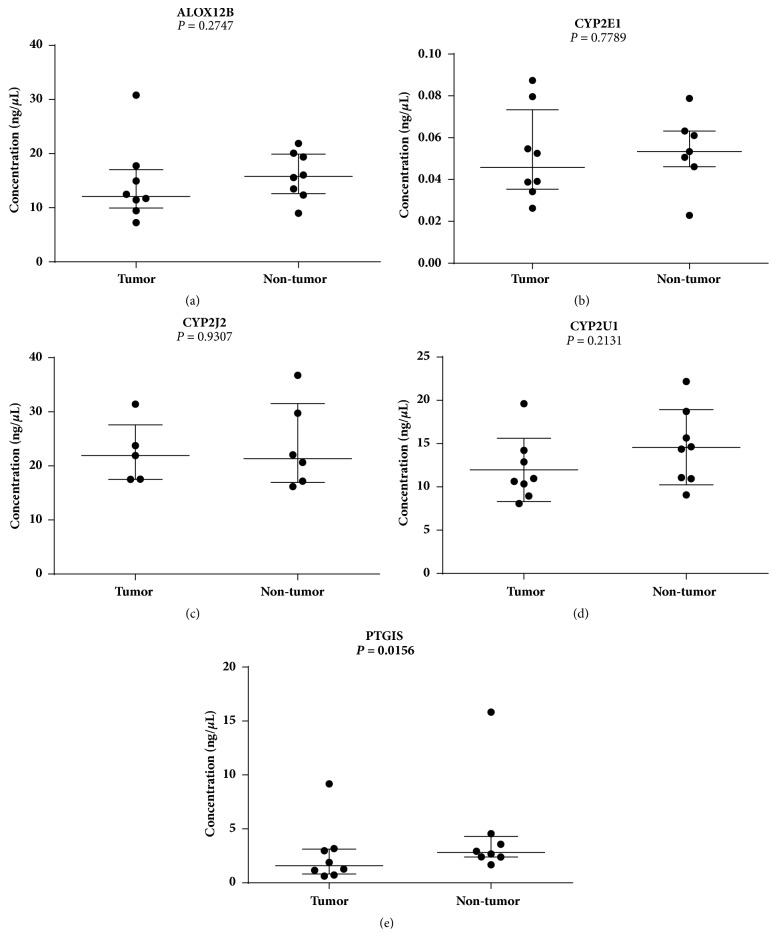
Protein expression by ELISA assay in oral tumors compared to non-tumor tissues. (a), (b) and (c) Results of Mann Whitney test (median with interquartile variation). (d) Results of Unpaired T test (mean with standard deviation). (e) Results of Wilcoxon matched paired test (median with interquartile variation), in bold the statistically significant p value. The concentration values of proteins are presented in linear scale.

The results of protein analysis by ELISA showed that expression of ALOX12 (median: OSCC = 12.09 ng/*μ*L vs. non-tumor = 15.80 ng/*μ*L), CYP2E1 (median: OSCC = 0.046 ng/*μ*L vs. non-tumor = 0.053 ng/*μ*L), CYP2J2 (median: OSCC = 21.91 ng/*μ*L vs. non-tumor = 21.34 ng/*μ*L), and CYP2U1 (mean: OSCC = 11.95 ng/*μ*L vs. non-tumor = 14.57 ng/*μ*L) proteins were not statistically different between OSCC and non-tumor tissues (p > 0.05), although the lower expression was observed in tumors. PTGIS protein showed significantly reduced expression in OSCC (median: OSCC = 1.58 ng/*μ*L vs. non-tumor = 2.80 ng/*μ*L; p = 0.0156) (Figure 3).

Expression of* ALOX12, CYP2E1*,* CYP2J2*,* CYP2U1, *and* PTGIS* genes were not correlated to expression of the respective proteins in OSCC (p = 0.243; p = 0.8397; p = 0.95; p = 0.4256 e p = 0.2430, respectively).

**Figure 4:**
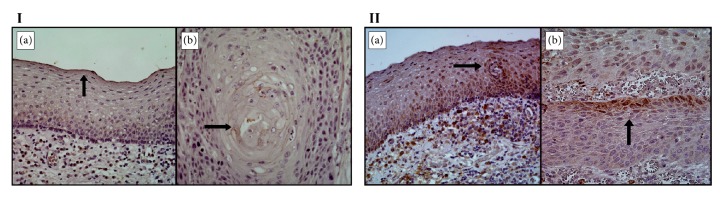
Analysis of ALOX12B and PTGIS proteins expression in tumor and non-tumor tissue by Immunohistochemistry.** I**: Expression of ALOX12B protein; the arrows show weak labeling (brown staining) of non-tumor squamous cells in (a) and OSCC cells in (b).** II**: Expression of PTGIS protein; the arrows show moderate labeling (brown staining) of non-tumor squamous cells in (a) and weak labeling of OSCC cells in (b).

IHC results showed that ALOX12B and PTGIS proteins presented low expression in groups of OSCC and non-tumor tissues (Figure 4).

Regarding clinical and histopathological parameters of tumor we did not find statistical significance between reduced expression of* CYP27A1*,* CYP2E1*,* CYP2R1*,* CYP2J2*,* CYPU1, CYP4F12*,* CYP4X1*,* CYP4B1, PTGIS, ALOX12,* and* MAOB* genes and increased expression of* CYP27B1* gene with tumor extension, nodal metastasis, and tumor progression in OSCC development.

## 4. Discussion

In the present study, differential expression was observed for 12 genes in OSCC compared to adjacent non-tumor tissues.* CYP27A1, CYP2E1, CYP2R1, CYP2J2, CYP2U1, CYP4F12, CYP4X1, CYP4B1, PTGIS *or* CYP8A1, ALOX12, *and* MAOB *genes presented reduced expression, while the* CYP27B1* gene showed increased expression in OSCC. Of the 24 pathways in which the enzymes encoded by these genes participate, 12 have been associated with carcinogenesis.

Differentially expressed genes encode enzymes of the cytochrome 450 monooxygenases (CYP450) family and other oxygenases as arachidonate lipoxygenase (ALOX) and monoamine oxidase (MAO). These enzymes play a role in oxidation reactions by adding one or more hydroxyl or oxygen molecules into the protein, lipid, or other ligand [50]. According to the literature, alteration in expression of these enzymes can be associated with oral carcinogenesis [30, 55, 56].

The metabolic pathways, in which the enzymes encoded by differentially expressed genes participate, can be involved in carcinogenesis highlighting the arachidonic acid metabolism (AA), the only pathway in which the protein encoded by the* PTGIS* gene (that remained significant after correction for multiple tests) participates.

In the AA metabolic pathway, PTGIS or CYP8A1 enzymes play a role catalyzing the conversion of prostaglandin H2 into prostacyclin (prostaglandin I2) [49]. The prostaglandin metabolic pathways have been involved in the inflammatory response [57] of important processes for the development of different types of cancers [58] by AA, a polyunsaturated fatty acid (PUFA) [59]. The AA, when oxygenated, is transformed into products that mediate or modify inflammatory reactions [59], and thus, it can activate or inhibit other pathways related to carcinogenesis [24, 60]. In addition to inflammation, production of metabolites derived from PTGIS-mediated reactions regulate physiological processes including angiogenesis, coagulation, proliferation, and immune response [24, 57, 61].

Studies have shown that the PTGIS enzyme is associated with the progression of cancer [61, 62]. Regarding the head and neck squamous-cell carcinoma type (HNSCC), a study has investigated carbaprostacyclin (cPGI_2_), a stable analogue of PTGIS, and showed little effect of analogue in cell migration of the HNSCC cell line. Furthermore, the authors suggested that the effect of PTGIS was related to its ability to promote vascularization; also, PTGIS gene and protein expression were shown to be reduced in HNSCC samples compared to non-tumor mucosa [63].

Our findings regarding reduced expression of the PTGIS gene and protein in patients with OSCC corroborate Camacho et al., who showed that this pathway could have contributed to the oral carcinogenesis in the present study. Reduction of PTGIS expression can modulate its possible anti-tumor functions [61] and contribute to carcinogenesis.

In addition to* PTGIS*, other genes of the CYP450 superfamily are involved in the AA metabolism, such as* CYP2E1, CYP2J2, CYP2U1 *(KEGG, 2015),* CYP4F12 *[52], and* CYP4B1* [64] genes. Cyclooxygenase (COX), arachidonate lipoxygenase (ALOX), and CYP450 epoxygenases enzymes use the AA as a primary precursor, generating eicosanoids including prostaglandins, leukotrienes, epoxyeicosatrienoic acids (EETs), and hydroperoxyeicosatetraenic acids (HPETEs) [65].

In this metabolism, CYP2E1, CYP2J2, and CYP2U1 enzymes generate 19-HPETE, CYP2J2 generates EETs, and CYP4F12 converts arachidonic acid to a cis-EET and dihydroxyeicosatrienoic acids (DHET) [53]. In addition, rabbit CYP4B1 has been shown to generate 12(R)-hydroxyeicosatetraenoic acid (12(R)-HETE) and 12-hydroxyeicosatrienoic acid (12-HETrE), inflammatory mediators, from arachidonic acid in an NADPH-dependent manner [64]. Although the role of CYP4B1 is unclear in humans, in the cancer community, there is a potential therapeutic strategy involving prodrug activation by the CYP4B1 transgene [66].

ALOX12 enzyme also participates in AA metabolism, and it regulates biological processes including platelet activation, angiogenesis induced by* Vascular Endothelial Growth Factor *(*VEGF*), apoptosis promoting the survival of vascular cells and control of cell migration and proliferation [49]. The ALOX12B enzyme can be procarcinogenic, as it converts AA in 12-HPETE and increases expression of encoding genes of proinflammatory cytokines such as Tumor Necrosis Factor (TNF-*α*) [67, 68].

Also acting within AA metabolism, ALOX12B and CYP2J2 enzymes perform the same function of generating 12-HPETE and EETs, respectively, from AA in the pathway of inflammatory mediator regulation of the Transient Receptor Potential (TRP) channels [47]. The TRP proteins of this pathway are cationic channels activated by 12-HPETE and EETs and belong to the molecular sensors superfamily that allows detection of environmental stimulus and promotes the senses [69]. The OSCC cell line has been observed with increased expression of* Transient Receptor Potencial Vanilloid* (*TRPV1*), which is activated by HPETE (a product of ALOX12B-mediated reactions). Furthermore,* TRPV1 *overexpression was associated with accelerated growth of these cells [70]. However, in patients with OSCC in our study, the* ALOX12B* gene showed reduced expression.

The metabolites derived from AA metabolism generated by CYP450, ALOX, and PTGIS also act, activating the Peroxisome Proliferator Activated Receptors (PPAR) [47]. This pathway promotes the signaling of transcription factors that regulate the expression of genes involved in lipid oxidation, inflammation, proliferation, and cell migration associated with tumorigenesis [71–73]. In this study, reduced expression of* CYP450*,* ALOX,* and* PTGIS* genes could be associated with the reduction of* CYP27A1 *gene expression in oral tumors investigated, since the PPAR activation promotes* CYP27A1 *gene expression.

CYP27A1 enzyme metabolizes cholesterol [47]. Cholesterol is derived from the vitamin D3-dependent steroid biosynthetic pathway, and it can be metabolized by the CYP27A1 enzyme in primary bile acid biosynthesis, used in steroid hormone biosynthesis, which also operates the CYP2E1 enzyme, or even directed to the degradation pathway of steroid [47].

Regarding primary bile acid biosynthesis, recent studies have revealed that the bile acid lithocholic type displays a significant cytotoxic effect on cancer cells in cultures. Thus, it has been related to cellular and molecular mechanisms of the antiaging and antitumor effects [74]. The reduced expression of the* CYP27A1 *gene observed in this study could lead to reduction in primary bile acid synthesis, reducing its antitumor effects.

CYPs play key roles in steroid biosynthesis, the pathway responsible for cholesterol synthesis. In this pathway, vitamin D3 is converted to calcidiol by the CYP2R1 enzyme and subsequently in calcitriol (1,25[OH]_2_D_3_), the circulating form of vitamin D3 [75], by the CYP27B1 enzyme [47]. Calcitriol can modulate the expression of the* CYP2R1* gene in OSCC, and thus the vitamin D analogs can be potential therapeutic agents in the control of OSCC progression [31]. In our study, the reduced expression of the* CYP2R1* gene in OSCC could be explained by vitamin D deficiency in patients with cancer, evidenced by literature [76–78], although vitamin D has not been quantified in these patients.

In addition to vitamin D, other steroid lipids from the steroid hormones biosynthesis [47], such as testosterone, androsterone, estrogen, and cortisone, can regulate cell signaling to control several physiological functions [47, 79].

Testosterone is formed from dehydroepiandrosterone (DHEA) in a CYP2E1-mediated reaction [47]. One study has shown an inhibitory potential of the DHEA compound on chemoprophylaxis to chemical carcinogens and mutations [80]. In our study, the reduced expression of the* CYP2E1 *gene could be related to OSCC development, possibly by reduction of DHEA and its function as an inhibitor of carcinogens.

Also acting in linoleic acid metabolism, the CYP2E1 and CYP2J2 enzymes use linoleate compound (linoleic acid) as a precursor to generate arachidonate, which can be directed to the metabolism of AA [47]. In our study, the reduced expression of* CYP2E1* and* CYP2J2* genes in OSCC could result in increased levels of linoleic acid. This is in agreement with the findings about the effect of diacylglycerol oil (composed of linoleic acid 46.6%) in transgenic rats carrying c-Ha-*ras* proto-oncogene human. This study showed that the increase of linoleic acid can promote oral cancer development [81].

In addition to catalyzing the oxidation of endogenous chemical components such as fatty acids (arachidonic-AA and linoleic), steroid hormones (testosterone), liposoluble vitamins (retinol and vitamin D3), and bile acid, CYP450 also metabolizes various chemical xenobiotics including drugs, environmental carcinogens, and amines [82].

In the oxidative biotransformation of xenobiotics, including drugs and other lipophilic exogenous compounds [83], CYP acts in Phase I, catalyzing the oxidation of substrates [84] and promoting metabolic activation of chemical carcinogens through the actions of the CYP2E1 [85], CYP4B1, and CYP4F12 enzymes [47, 52, 86]. Although our findings have demonstrated reduced expression of* CYP2E1, CYP4B1,* and* CYP4F12* genes in OSCC, we cannot report modification of their enzymatic activity, since even at low concentrations, as observed in this study, these enzymes can generate activated carcinogenic compounds.

In addition to CYP, the Monoamine Oxidase B enzyme (MAOB) also acts in drug metabolism. MAOB is also an important flavoenzyme in regulating the metabolic degradation of serotonin in nerve tissue or the target tissues [51]. MAOB acts, as well, in the serotonergic synapse, along with the ALOX12, CYP2J2, and CYP4X1 enzymes [47]. Decreased activity of MAO can interfere with the effects of antidepressant drugs in serotonergic neurotransmission [87] by means of increasing serotonin, which acts as a mediator of cell division [88]. The inhibition of this enzyme has been associated with increased serotonin levels and consequent increase of cell proliferation in colon tumors [89]. Our findings regarding MAOB-reduced expression in OSCC corroborate with the results obtained by Chen and et al., in pharyngeal cancer [90].

Another important MAOB-associated mechanism for carcinogenesis is the metabolism of amino acids such as tyrosine, glycine, serine, threonine, tryptophan, histidine, arginine, proline, and phenylalaninein [47]. Amino acids have a dual role in cellular metabolism, as precursors for protein synthesis and as intermediate metabolites in other biosynthetic reactions. Disruption of these processes is generally observed in cancer, and serine and glutamine are the most common amino acids used by tumor cells [91]. Amino acids are used in the machinery of mTOR (mechanistic target of rapamycin), which is also altered in cancer [78]. The reduced expression pattern of the MAOB gene in this study could be associated with alteration in the metabolism of amino acids as well as influencing the machinery mTOR and thereby contributing to development of OSCC.

MAOB also acts by catalyzing the oxidative deamination of biogenic monoamines and exogenous compounds [49, 92], generating hydrogen peroxide (H_2_O_2_) and acetaldehyde, which are able to induce cell death in cultures of various human tumor cell lines. H_2_O_2_ can directly interact with molecules and endogenous structures, resulting in oxidative stress [92]. In addition to MAOB, CYP450 is also involved in oxidative stress [9] and production of reactive oxygen species (ROS) [93–95], which can lead to DNA damage and mutation, resulting in proto-oncogene activation or tumor suppressor genes inactivation and promoting cancer [96]. In addition, an oxidative/nitrosative stress study suggested that it could play a significant role in oral cavity cancer and that curative resection is effective in alleviating this oxidative/nitrosative burden, since tumors form the major source of oxidants [97].

ROS are derived from the consumption of a nicotinamide adenine dinucleotide phosphate (NADPH) cofactor by CYP450 microsomal [24, 96, 98, 99]. In steroid biosynthesis, CYP2R1 and CYP27B1 enzymes can regulate oxidative stress by calcitriol synthesis, which acts as a hormone interfering in levels of ROS through regulation of the expression of regulator genes belonging to the antioxidant system [78].

Furthermore, evidence indicates that ROS production induces* VEGF-A* (*Vascular endothelial growth factor *A) gene expression [100], related to angiogenesis, and can promote the metastatic growth of tumor cells [101]. Moreover, VEGF is necessary to transport polyunsaturated fatty acids to endothelial cells. Thus, a decrease in VEGF levels can reduce the formation of anti-inflammatory and angiogenic factors, which participate in processes such as oxidative stress, endothelial dysfunction, insulin resistance, and production of prostacyclin [102] (generated by PTGIS), an important factor in endothelial vasodilation [103].

Therefore, reduced expression of genes encoding MAOB, CYP2E1 CYP2R1, PTGIS, and CYP27B1, which are involved in oxidative stress, could modulate the intracellular balance of ROS/antioxidants and the risk for OSCC development.

## 5. Conclusions

The differential expression pattern of the studied genes could modulate metabolisms that contribute to oral squamous cell carcinoma development. These metabolisms are involved in processes such as inflammation, inhibition of carcinogenic agents, lipid oxidation, oxidative stress, autophagy, apoptosis, cell differentiation and proliferation, tumorigenesis, angiogenesis, and vasodilation, which contribute to migration and invasion of cancer cells in tissues of different histological type, promoting metastasis. An important metabolic pathway involved in the OSCC tumorigenesis evidenced in this study is the arachidonic acid metabolism, in which the PTGIS enzyme participates, presenting more significant differential expression in tumors. Further investigations, with expansion of the sample group, are necessary to establish the relationship between the expression pattern of the investigated genes and oral squamous cell carcinoma.

## Figures and Tables

**Table 1:** Differential gene expression in OSCC compared to adjacent non-tumor tissue.

**Gene**	**Gene name**	**Gene ID** ^**a**^					
***One Sample T test***			**Mean RQ**	**Std Dev** ${}^{\overset{\;}{b}}$	**p value**	**p' value** ^**∗**^	**p – p'** ^**∗∗**^
*CYP27A1*	*Cytochrome P450, Family 27, Subfamily A, Polypeptide 1*	1593	0.4952	0.3721	0.0064	0.00323	0.003174
*CYP2E1*	*Cytochrome P450, Family 2, Subfamily E, Polypeptide 1 *	1571	0.5111	0.387	0.0091	0.00484	0.004261
*CYP2R1*	*Cytochrome P450, Family 2, Subfamily R, Polypeptide 1*	120227	0.5472	0.4618	0.0276	0.00726	0.020342
*CYP2J2*	*Cytochrome P450, Family 2, Subfamily J, Polypeptide 2*	1573	0.4178	0.2963	0.0009	0.00081	0.000094
*CYP2U1*	*Cytochrome P450, Family 2, Subfamily U, Polypeptide 1*	113612	0.6086	0.4472	0.0425	0.00968	0.032823
*CYP4F12*	*Cytochrome P450, Family 4, Subfamily F, Polypeptide 12*	66002	0.4057	0.417	0.005	0.00242	0.002581
*CYP4X1*	*Cytochrome P450, Family 4, Subfamily X, Polypeptide 1*	260293	0.4639	0.4158	0.0082	0.00403	0.004168
*PTGIS/ CYP8A1*	*Prostaglandin I2 (Prostacyclin) Synthase / Cytochrome P450, Family 8, Subfamily A, Polypeptide 1*	5740	0.3479	0.3419	0.001	0.00161	**-0.000613**
***Wilcoxon Signed Rank Test***			**Median RQ**	**75th per** ^**c**^	**p value**	**p' value** ^**∗**^	p – p'^**∗∗**^
*ALOX12*	*Arachidonate 12-Lipoxygenase*	239	0.03578	0.2277	0.0391	0.00806	0.031035
*CYP27B1*	*Cytochrome P450, Family 27, Subfamily B, Polypeptide 1*	1594	1.927	6.343	0.0391	0.00887	0.030229
*CYP4B1*	*Cytochrome P450, Family 4, Subfamily B, Polypeptide 1*	1580	0.08512	0.618	0.0234	0.00645	0.016948
*MAOB*	*Monoamine Oxidase B*	4129	0.1882	0.3693	0.0156	0.00565	0.009955

^a^Gene Identification second-base gene data, the National Center for Biotechnology Information (NCBI, http://www.ncbi.nlm.nih.gov/gene); ^b^Standard Deviation; ^c^75th Percentile; ^*∗*^p' value after Benjamini-Hochberg False Discovery Rate; ^*∗∗*^p-p' Statistical significance occurs when the p value of the individual tests is less than the p' value after correction for multiple tests. Significance is only confirmed for negative result of the subtraction of these values (value p - value p'), emphasized in bold.

**Table 2:** The metabolic pathways of Human species related to the 12 differentially expressed genes in OSCC.

**Human metabolic pathways**	**Genes **
Metabolism of xenobiotics by cytochrome P450	CYP2E1, CYP4B1 and CYP4F12

Chemical carcinogenesis	CYP2E1 and CYP4B1

Drug metabolism by cytochrome P450	CYP2E1, MAOB, CYP4B1 and CYP4F12

Serotonergic synapse	ALOX12, CYP2J2, CYP4X1 and MAOB

Arachidonic acid metabolism	ALOX12, CYP2E1, CYP2J2, CYP2U1, CYP4F12 and PTGIS

Inflammatory mediator regulation of TRP^a^ channels	ALOX12 and CYP2J2

PPAR^b^ signaling pathway	CYP27A1

Steroid biosynthesis	CYP2R1 and CYP27B1

Steroid hormone biosynthesis	CYP2E1

Primary bile acid biosynthesis	CYP27A1

Linoleic acid metabolism	CYP2E1 and CYP2J2

Amino acids metabolism^c^	MAOB

Ovarian steroidogenesis	CYP2J2

Tuberculosis	CYP27B1

Nonalcoholic fatty liver disease (NAFLD)	CYP2E1

Dopaminergic synapse	MAOB

Amphetamine, cocaine, and alcohol addiction	MAOB

^a^TRP: Transient Receptor Potential; ^b^PPAR: Peroxisome Proliferator Activated Receptors. ^c^The amino acids include glycine, serine, threonine, tyrosine, tryptophan, histidine, arginine, proline, and phenylalanine.

## Data Availability

All data generated or analysed during this study are included in this published article; if necessary the datasets used and/or analysed during the current study are available from the corresponding author on reasonable request.
